# The Solution for the Thermographic Measurement of the Temperature of a Small Object

**DOI:** 10.3390/s21155000

**Published:** 2021-07-23

**Authors:** Arkadiusz Hulewicz, Krzysztof Dziarski, Grzegorz Dombek

**Affiliations:** 1 Institute of Electrical Engineering and Electronics, Poznan University of Technology, Piotrowo 3A, 60-965 Poznan, Poland; 2 Institute of Electric Power Engineering, Poznan University of Technology, Piotrowo 3A, 60-965 Poznan, Poland; krzysztof.dziarski@put.poznan.pl (K.D.); grzegorz.dombek@put.poznan.pl (G.D.)

**Keywords:** thermography, programmable logic controllers, thermographic camera, microscopic thermography

## Abstract

This article describes the measuring system and the influence of selected factors on the accuracy of thermographic temperature measurement using a macrolens. This method enables thermographic measurement of the temperature of a small object with an area of square millimeters as, e.g., electronic elements. Damage to electronic components is often preceded by a rise in temperature, and an effective way to diagnose such components is the use of a thermographic camera. The ability to diagnose a device under full load makes thermography a very practical method that allows us to assess the condition of the device during operation. The accuracy of such a measurement depends on the conditions in which it is carried out. The incorrect selection of at least one parameter compensating the influence of the factor occurring during the measurement may cause the indicated value to differ from the correct value. This paper presents the basic issues linked to thermographic measurements and highlights the sources of errors. A measuring stand which enables the assessment of the influence of selected factors on the accuracy of thermographic measurement of electronic elements with the use of a macrolens is presented.

## 1. Introduction

In an era of intense technological development, an increasing number of electronic devices and their miniaturization, the diagnostics of such devices, understood as cyclical monitoring, the assessment of proper operation and, in the case of detection of irregularities, the identification of the source of the problem, plays an important role [1]. Early diagnosis of irregularities in the operation of individual electronic components allows users to avoid damaging the entire device and minimizes repair costs. Since damage to these elements is often preceded by a rise in temperature [2], an effective method is the monitoring of the temperature distribution on the surface of the components of the observed device. Various studies have considered a thermographic camera as a good tool to measure component temperatures [3,4]. Thermographic cameras record the infrared radiation emitted by the observed object. Thermography is a noncontact method, which facilitates the measurements, shortens their duration and causes the method to be safe [3]. Depending on the range of IR radiation, thermographic cameras use detectors that ensure maximum sensitivity in the SWIR (Short Wave InfraRed) band with a wavelength of *λ* = 1–2 µm, in the MWIR band (Medium Wave InfraRed) with *λ* = 2–5 µm and LWIR (Long Wave InfraRed) with *λ* = 7–14 µm [3].

Thermographic measurements of electronic devices deserve special attention. In the case of thermographic diagnostics of small-sized elements, such as SMD (Surface Mounted Device) or BGA (Ball Grid Array), it is required to use an additional wide-angle macrolens [2,5]. It makes it possible to evaluate temperature distribution on the housings of the abovementioned electronic components and changes in its value over time. The analysis of the recorded temperature distributions and temperature changes over time allows us to detect possible threats that may occur at a later time. Moreover, a correctly registered temperature distribution on the casing enables the estimation of the temperature of the semiconductor junction of the tested electronic element [3].

In order to correctly assess temperature distribution on the housing of electronic components, it is necessary to take into account all factors that can influence the measurement [6,7,8]. The most important of them include the value of the emissivity coefficient *ε* [9], the reflected temperature *ϑ_r_* [10], the distance between the lens and the observed object *d* [11], the ambient temperature *ϑ_a_* [12], the temperature of the external optical system [13], the transmittance of the external optical system [14,15], the relative humidity ω [16], the viewing angle *β* [7], and the sharpness of the recorded thermogram [17,18,19]. An assessment of the influence of the abovementioned factors on the accuracy of the thermographic temperature measurement of small-sized electronic components is possible thanks to the use of a microscopic thermography stand. Depending on the conditions in which the measurement is performed, the influence of individual factors on the result of the thermographic temperature measurement will differ [19]. Some parameters will have a greater effect when the value of *d* is large (kilometers) and other will have a greater impact in microscopic thermography (*d* measured in millimeters). In the case of microscopic thermography, particular attention should be paid to the selection of the *ε* value, which is the ratio of the power of radiation emitted by the observed surface and the power of radiation emitted by a perfect black body at the same temperature *ϑ* and the same *β* (1) [20].

(1) $$\frac{m\left(\lambda ,\vartheta \right)}{m_{c}\left(\lambda ,\vartheta \right)}=\varepsilon \left(\lambda ,\vartheta \right)$$

where *m* (*λ*, *ϑ*) is the existence (radiation power density) of the surface of the tested body, and *m_c_* (*λ*, *T*) is the existence of a black body.

In the measurement, the value of *ε* should be chosen that is closest to *ε* of the real value of the observed surface [6,7,9]. The lower the accuracy with which *ε* is chosen, the lower the accuracy of the thermographic temperature measurement will be. Another parameter that is particularly important in microscopic thermography is *d*. This parameter is related to the transmittance of the atmosphere *τ_a_*. The value of *τ_a_* changes when the values of *d* and *ω* [21] change. The lower the value of *τ_a_*, the less IR radiation reaches the IR detector array of the thermographic camera. Consequently, due to the lower power of IR radiation absorbed by the array of IR detectors, the amplitude value of the output detector signal is lower [14,15,16,17]. In the case of microscopic thermography, due to the small values of *d*, the values of *τ_a_* and *ω* have little influence on the indication of the thermographic camera [19]. The value of *d* is important in microscopic thermography, because due to the shallow depth of field of the lenses used, even a slight change in *d* (in the order of a tenth of a millimeter) changes the sharpness of the recorded thermogram [18]. Another way to change the sharpness of the recorded thermogram is to change the focusing ring position angle [17]. As a result of the conducted research, it has been confirmed that in the case of thermograms classified as sharp, the error value of the temperature measurement is lower than in the case of thermograms classified as out-of-focus. Taking into account the unsharpness of the thermogram increases the reliability of diagnosing potential damage to electronic devices caused by the temperature increase in electronic components [17,19].

In normal operation, most electronic components reach a certain temperature value, which can be monitored by analyzing thermograms taken at specific intervals [11]. In the case of damage to a specific component, its temperature changes, which is reflected in the recorded thermogram [22,23,24]. Correct diagnosis of such damage to equipment is possible only if the obtained thermograms are correctly interpreted and the influence of the disturbing factors is taken into account in advance. For this reason, a stand that would minimize the impact of these factors on the measurement result has been proposed. While analyzing the commercial solutions of microscopic thermography stands that enable thermographic temperature measurement of electronic components, the described solution was not found [25,26,27]. None of the available stands ensures sufficient optical isolation of the observed object from sources of IR radiation coming from outside the chamber. In addition, none of the offered stands provides continuous measurement of the factors influencing the result of thermographic temperature measurement. This allows us to change the settings of the IR camera in order to minimize the influence of these factors on the result of thermographic temperature measurement. Moreover, the possibility of precise and repeatable setting of the distance *d* (camera lens-observed object) *d* and the observation angle *β* was not encountered. Most stands for thermographic observation of small-size electronic components are in the form of an open table, as a result of which it is not possible to ensure a proper optical isolation of the measuring system and to minimize reflections from the system components [25,26,27]. This article describes a stand, which allows us to determine the share of factors influencing the accuracy of the temperature measurement of electronic components with small dimensions. The assumptions related to the prepared stand and the results of the research obtained with its use have been presented. These assumptions take into account all aspects that have not been used in commercial solutions so far. The results of the conducted research allowed us to verify the adopted assumptions and present the practical application of the stand.

## 2. Materials and Methods

### 2.1. The Measurement Stand

The most important elements of the constructed stand are the measurement chamber, a thermographic camera with a macrolens and an automatic regulation system, whose task is to control the parameters inside the chamber and maintain them at a given level. The controlling element of the automatic control system is a programmable logic controller (PLC) and an HMI (Human-Maschine Interface) touch panel cooperating with it. The main assumption of the constructed stand was to ensure repeatable and stable values of the parameters important in microscopic thermography. The use of a PLC as an element for controlling and measuring the parameters inside the measuring chamber is another example of the unconventional application of the stand [28,29,30,31].

The PLC controller in combination with a specific set of sensors and the HMI operator panel ensures the effective control of the measuring chamber parameters, while maintaining their repeatability and stability. In addition, it allows us to create any settings. The management system presented in this article uses a programmable logic controller (PLC), an operating panel (HMI), a set of stepper motors and selected sensors. Based on the constructed system, an analysis of its functionality was carried out, which made it possible to prove that the solution using a PLC controller allows us to achieve satisfactory parameters, while reducing installation and operation costs.

The Flir E50 thermographic camera (Flir, Wilsonville, OR, USA) [32] with an additional Close up 2x lens (Flir, Wilsonville, OR, USA) [33] was placed in the chamber, which was part of the constructed microscopic thermography stand. The accurancy of the thermographic camera is equal to ±2 °C (±3.6 °F) or ±2% of the reading, for ambient temperatures of 10 °C to 35 °C (+50 °F to 95 °F) [32]. The camera was placed on a linear module driven by a stepper motor [34]. This enables the *d* value to be changed. On this basis, it is possible to evaluate the influence of the *d* value and sharpness on the accuracy of the thermographic temperature measurement.

**Figure 1:**
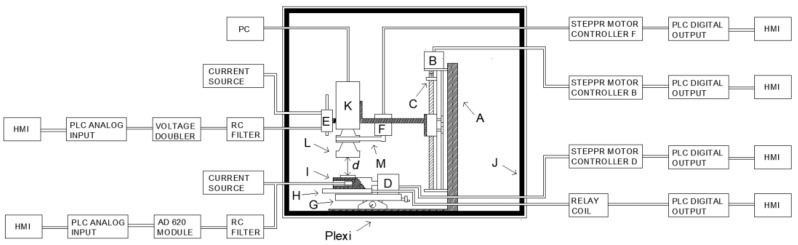
View of the schematic of measurement system: (A) Tripod; (B) Stepper motor; (C) Linear guide; (D) Stepper motor; (E) Resistance linear distance sensor; (F) Stepper motor; (G) Cross table; (H) Heating mat; (I) Aluminium bloc with Pt1000 temperature sensor; (J) Polyurethane foam; (K) Thermographic camera lens; (L) Additional macrolens; (M) Close-up lens 2× P/NT 197,200 (d) WD (Work Distance)—distance between observed object and thermographic camera macrolens.

In addition, the influence of image sharpness on the measurement result can be judged from the α settings. This change is carried out by means of a stepper motor connected to the lens with an elastic strap. In order to assess the influence of the viewing angle on the accuracy of the thermographic measurements, a dedicated base was used, on which the tested electronic elements were placed. This base is connected with a stepper motor that allows us to change its position angle β in relation to the camera lens. A heating element was installed in the aforementioned base, whose task was to keep the set temperature of the observed component constant. In addition to the actuators in the chamber, there are also the measuring systems. These systems make it possible to measure the temperature of the observed component with the contact method and to measure the *d* value. All measuring and actuating elements, as well as the PLC controller and the thermal-vision camera were placed on the measuring stand in accordance with the block diagram shown in Figure 1.

### 2.2. Components

In the presented measuring system, the control component was a programmable logic controller (PLC). From among the available controllers, the Siemens Si-matic S7-1200 controller, version CPU 1214 DC/DC/DC was selected, designated as 6ES7214-1BG40-0XB0 (Siemens AG, Munich, Germany) [35]. It allows us to control and monitor fast-changing processes with the use of transistor outputs. Its biggest advantages include autonomous work and cooperation with visualization components. In this controller, there are 14 digital inputs and 10 outputs, signaling LEDs and a communication connector used for programming the controller and communication with other external devices (the central unit uses the built-in PROFINET interface and communication takes place via a network cable connected via connector RJ45). It also has two analog inputs (in the voltage range from 0 to 10 V) and one voltage output (in the voltage range from 0 to 10 V and current from 0 to 20 mA) [35].

The controller works directly with the HMI panel Siemens Simatic KTP 600 Basic PN (Siemens AG, Munich, Germany) [36]. The panel is equipped with a 6-inch color display and a touch screen. The display is made in the LCD TFT technology with 256 colors. The resolution of the touch screen is 240 × 320 pixels. There are 6 freely configurable buttons under the touchscreen. It is used primarily in medium-sized control systems and most often works with the Siemens Simatic S7-1200 controller. The user memory is 1 MB and the configuration software is WinCC Basic [37]. The communication with the controller is run via the RJ45 Ethernet connector. The use of the operator panel connected with the PLC controller enables the setting of the parameters of the stepper motors and controlling their position. The buttons used allow us to switch on the selected stepper motor and select the direction of its rotation. In the area of controlling the stepper motor, it is also possible to select manual and automatic control.

**Figure 2:**
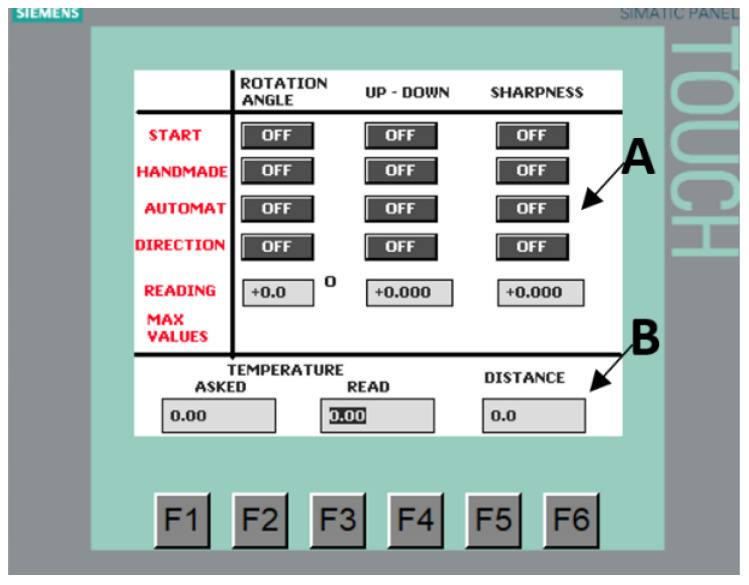
View of the touch panel window; A—area for controlling stepper motors; B—area of measurement fields.

For safety reasons, restrictions preventing the limit position of the settings of the controlled stepper motors from being exceeded and the reading fields which visualize the current position of these settings have been added. Moreover, the panel makes it possible to set the temperature of the component observed by the camera, read the contact temperature measurement of this component as well as the distance between the observed component and the lens of the thermographic camera (Figure 2).

**Figure 3:**
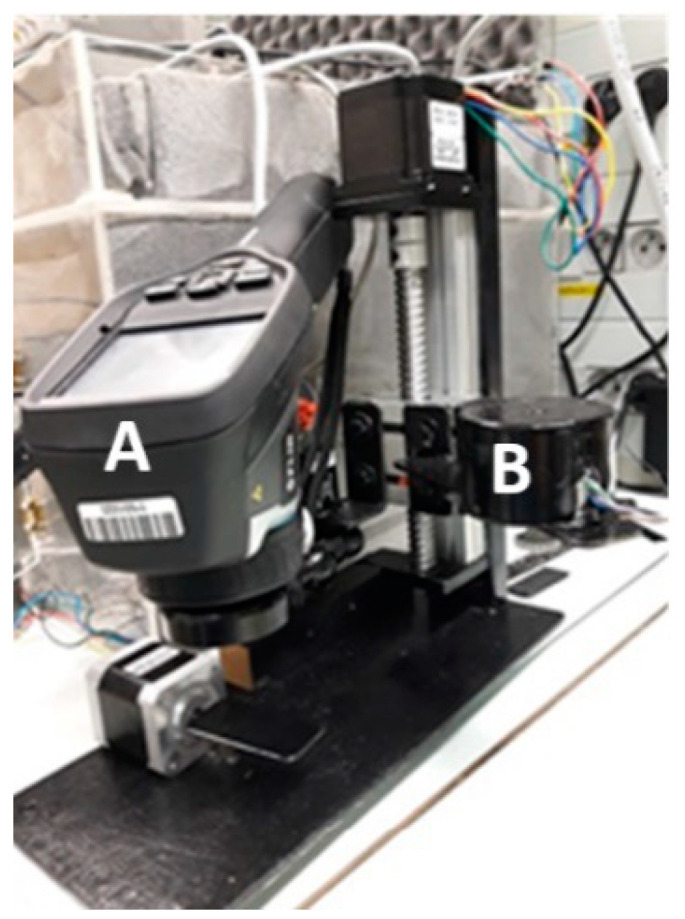
View of the circuit for adjusting the angle of the focus ring (*α_k_*): A—thermographic camera with close up 2× lens; B—stepper motor to adjust the angle *α_k_*.

Obtaining the distance between the thermographic camera lens and the observed component as set on the touch panel, as well as the position of the angle of the IR camera lens ring and the position angle of the observed component in relation to the camera lens, was achieved by means of stepper motors appropriately connected with the PLC controller. A unipolar stepper motor connected to the IR camera lens with an elastic strap was used to adjust the angle of the focusing ring of the thermographic camera (α), which directly influences the sharpness of the observed image. The motor parameters allow us to change the angle of the thermographic camera ring with a step α*_k_* = 1.5°, in the range from 0 to 45°. Information about the current value of the angle α*_k_* is displayed on the HMI panel (Figure 3).

**Figure 4:**
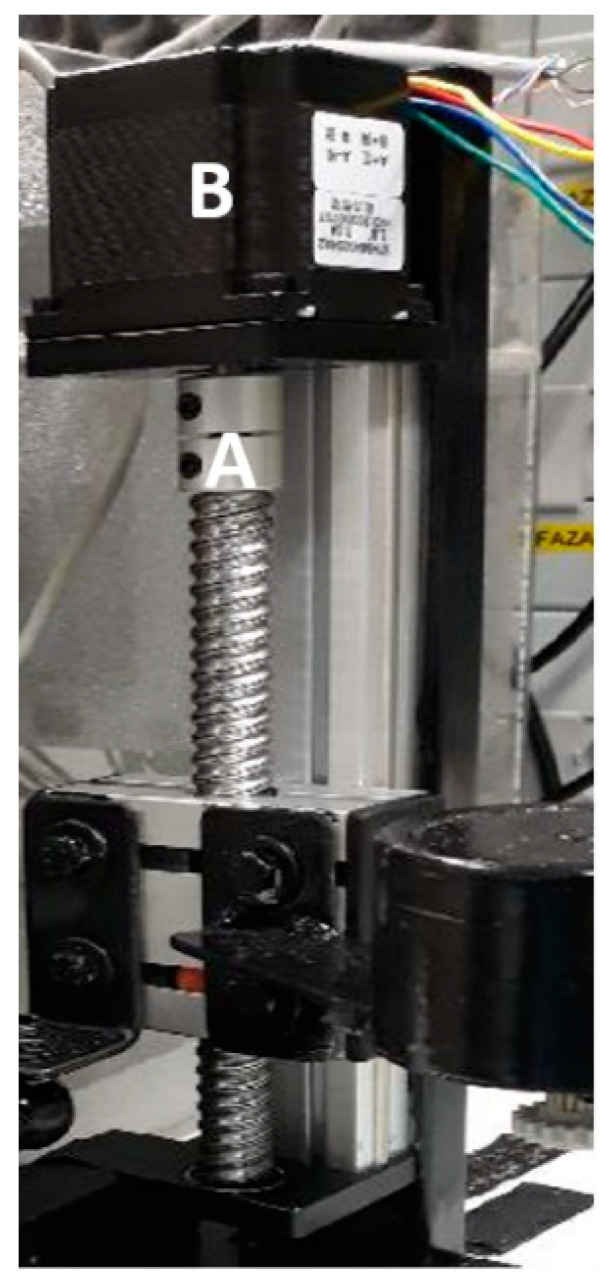
View of the distance adjustment circuit *d* (lens-observed component); A—worm gear; B—stepper motor to adjust the distance between the lens and the observed element.

In order to assess the impact of changes in distance *d* between the IR camera lens and the observed component, a linear module was used, where a thermographic camera was mounted. This module is equipped with a worm gear driven by a bipolar stepper motor marked with the symbol 57H56H3004A2 (Figure 4) [34], connected to a PLC controller. The applied driver and algorithm make it possible to change the position of the camera lens distance in relation to the observed object of the component with a step of 0.1 mm. As in the previous case, information about the current lens displacement of the observed component is displayed on the HMI panel.

**Figure 5:**
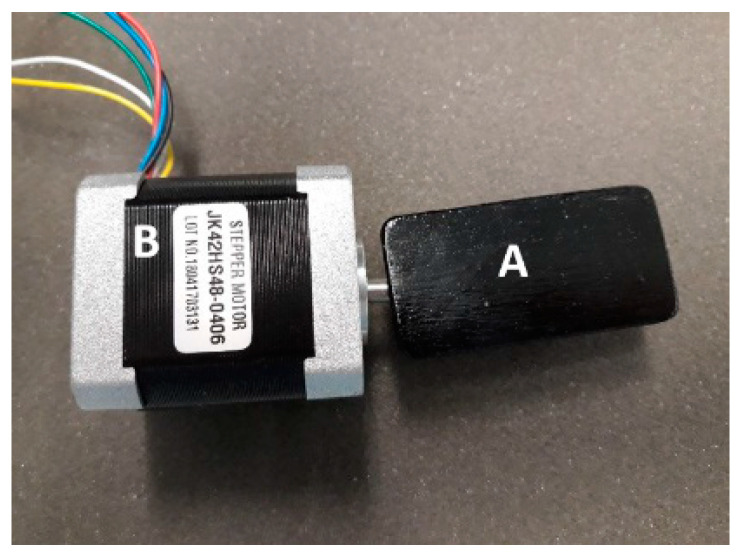
View of the *β* observation angle adjustment circuit: A—stand for the tested component; B—stepper motor to adjust the observation angle *β*.

Apart from the sharpness of the image recorded with the camera, the observation angle of the tested element *β* has a significant impact on the accuracy of temperature measurements in thermographic microscopy. In order to verify the influence of this angle on the temperature measurement error, the presented measurement stand was equipped with a specialist stand integrated with the stepper motor (Figure 5). The motor, controlled by a PLC controller, allows us to change the position angle *β* of the abovementioned stand in the range from −90° to +90° in a 0.9° step.

In addition to the presented control systems, the constructed stand was equipped with a measuring system allowing us to determine the temperature of the tested component and the distance *d* between the tested component and the lens of the thermographic camera. A system measuring this distance was integrated with the already discussed system allowing us to change the value of d. In this system, the said quantity was measured with the MMR30 potentiometric pickoff sensor with a nominal resistance value of 10 kΩ [38]. This sensor made it possible to measure the distance *d* in the range of 30 mm with a resolution equal to 0.1 mm. These parameters meet the requirements for close-up measurements. According to the data of the Close-up lens manufacturer’s datasheet, a sharp thermogram can be obtained when the distance *d* = WD = 33 mm. If this distance is kept, a sharp thermogram can be obtained and the spatial resolution is known. On the other hand, the greatest permissible difference between *d* and WD is a value equal to 0.4 mm. This value is greater than the declared resolution of the sensor used, therefore, in the presented test stand, it is possible to carry out measurements with sufficiently good precision.

### 2.3. Measurement Systems

**Figure 6:**
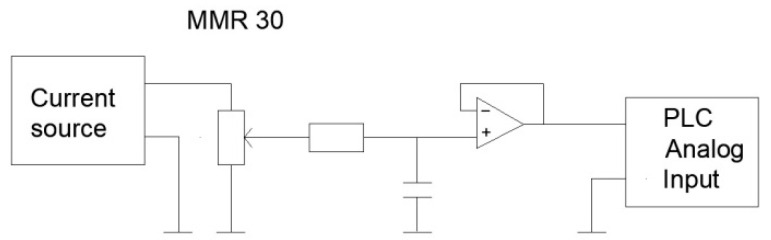
Diagram of the processing path of the measurement system *d (d*—the distance between the lens and the observed object).

Acquisition of measurements from the sensor was possible after its previous supply with a current signal of a low intensity value from a current source connected to the Howland circuit [39]. The signal obtained from the sensor was fed to the analog input of the PLC controller. Due to the potentiometric nature of the sensor and the low value of the PLC input resistance, an operational amplifier was used in the measurement circuit, connected in a voltage follower configuration. Moreover, the low value voltage was susceptible to interference from the power grid and stepper motors working in the test stand. These interferences significantly influenced the stability of the final measurement result. In order to minimize them, capacitors were used at the current source and the sensor, and an RC filter was applied in the input circuit of the PLC controller (Figure 6).

The whole set is connected with the use of a shielded FTP cable. The voltage signal presented on the HMI panel. The displayed information provides full control of the distance between the observed electronic component and the lens of the thermographic camera during its changes.

**Figure 7:**
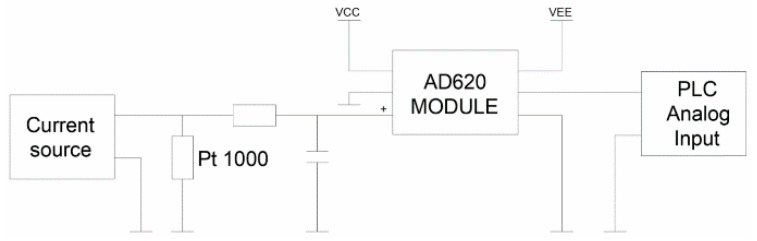
Diagram of the processing path of the temperature measurement system.

Apart from the measurement of d, the temperature of the tested component is also controlled in the constructed stand. It is monitored in parallel with the thermographic measurements and is it carried out using the PT1000 sensor [40]. According to the manufacturer’s catalog note, the sensor is powered from a current source connected to a Howland circuit with a current intensity of 100 μA. The low value of the received signal made it necessary to apply filtration, as in the previous system and, in addition, appropriate amplification of the acquired signal. For this purpose, the AD620 [41] amplifier working in the configuration of a differential amplifier was used. The gain value was adjusted to the range of the measured temperatures and the voltage range of the analog input of the controller. Moreover, the applied amplifier was equipped with an offset correction system (Figure 7). As before, the voltage signal obtained from the temperature sensor was appropriately scaled using the PLC software and presented on the HMI panel.

The presented temperature measurement system made it possible to build a control system that enables setting and maintaining a specific temperature of the tested component. This function is performed by the heating component coupled by temperature measurement software. In keeping with the adopted assumptions, the hysteresis of the set temperature was 2 °C. Apart from the abovementioned systems, the main component of the presented stand was the measuring chamber. The chamber is made of plexiglass, in the form of a cuboid sized 45 cm × 45 cm × 33 cm. The walls of the chamber are lined with black polyurethane foam. The foam used is of a porous structure and its single pore idealizes the black body model. Such a structure of the chamber is characterized by a high value of *ε* = 0.95 [42] and a small value of the reflectance factor *ρ*.

Moreover, the structure of this chamber ensures proper optical isolation of the measuring system and minimizes the reflections of IR radiation coming from the measuring system components. From among the abovementioned systems, a thermographic camera, sensors for distance and temperature measurement, stepper motors and the presented linear module were placed in the chamber. The actuators inside the chamber were blackened with thermographic paint. All the other components of the stand were placed outside the chamber in order to minimize undesirable interference factors. The software controlling the whole set has been archived on the website of the Poznan University of Technology under the identifier r1880_2021 [43].

### 2.4. The Research Methodology

In order to verify measurement correctness for the parameters whose values were displayed on the HMI screen placed on the stand, a series of tests was performed. During the tests, particular attention was paid to the reading accuracy of the set distances *d* and the correctness of the thermographic temperature measurement of the tested electronic component. Additionally, a series of measurements was carried out to determine the influence of the image sharpness and the observation angle *β* on the accuracy of the thermographic temperature measurement when the distance *d* was small (a few millimeters).

At the beginning, the correctness of the temperature measurement was checked by means of the PT1000 *ϑ_s_* sensor installed in the stand, on which the tested component was placed [40]. The value of the measured temperature was displayed on the HMI panel. After each measurement, the measured sensor temperature was determined on the basis of its resistance and the known function *ϑ*_s_ = f(*R*), where *R* is resistance of the Pt1000 sensor. The temperature of the stand measured with the tested sensor, was set in the range from 30 °C to 100 °C, in steps of 5 °C. Assuming the temperature value determined by function *ϑ_s_* = f(*R*) as the reference temperature, the error was determined on the basis of Formula (2) [44].

(2) $$\delta_{T}=\frac{\vartheta_{r}-\vartheta_{f}}{\vartheta_{f}}\times 100\%$$

where *δ_T_* is a temperature measurement error, *ϑ_r_* is a temperature read by means of a microscopic thermography stand, and *ϑ_f_* is a temperature determined by function *ϑ_s_* = f(*R*).

The correctness of the reading of distance *d* was also verified. For this purpose, the *d* measurement was performed with the use of the constructed stand and with the use of the FT50RLA-70-S1L8 laser distance sensor (SensoPart, Industriesensorik GmbH, Gottenheim, Germany) [45]. The sensor works on the basis of the triangulation method and enables measurements in the range from 30 mm to 100 mm, with a resolution of 0.1 mm. The measurement result was obtained on the basis of the 4–20 mA current signal. The distance was changed from 0 mm to 30 mm in 1 mm steps. Assuming the value of *d* measured with the FT50RLA-70-S1L8 sensor as the reference distance, the error was determined on the basis of Formula (3) [44].

(3) $$\delta =\frac{d_{r}-d_{s}}{d_{s}}\times 100\%$$

where *δ* is a distance measurement error, *d_r_* is a distance read by the microscope thermography stand, and *d_s_* is a distance read by the FT50RLA-70-S1L8 sensor.

In order to determine the influence of the sharpness of the recorded thermogram on the thermographic error of the temperature measurement, three series of thermograms were made showing the Pt1000 sensor in a round cover. During each series of measurements, the focus was changed using the angle of the position of the focusing ring sharpness α, with the constant distance value of *d* = 33 mm. The value of α was changed in 1.5° steps, ranging from 0° to 45°. The actual temperature of the observed sensor was read from the HMI panel and determined on the basis of function *ϑ_s_* = f(*R*). The obtained thermograms were presented to a group of 137 volunteers, whose task was to indicate the sharpest thermogram. The observers’ indications were compared with the results obtained using the mathematical measure of sharpness.

Finally, the correctness of the reading of angle *β* was verified. For this purpose, the stand (A—Figure 5) was changed to an aluminum block, whose upper surface was painted with Velvet Coating 811-21 paint with a known ε value ranging from 0.970 to 0.975 for temperatures ranging from −36 °C to 82 °C. The uncertainty with which the value of ε was determined was 0.004 [46]. The temperature of the block was measured with a PT1000 sensor located in the block. The β value is the angle between the top surface of the aluminum block and the plane in which the lens was located. The *β* value was changed from 0–90° in steps of 0.9°. For each value of the angle, a thermographic measurement of the temperature of block surface covered with paint was read.

## 3. Results

**Figure 8:**
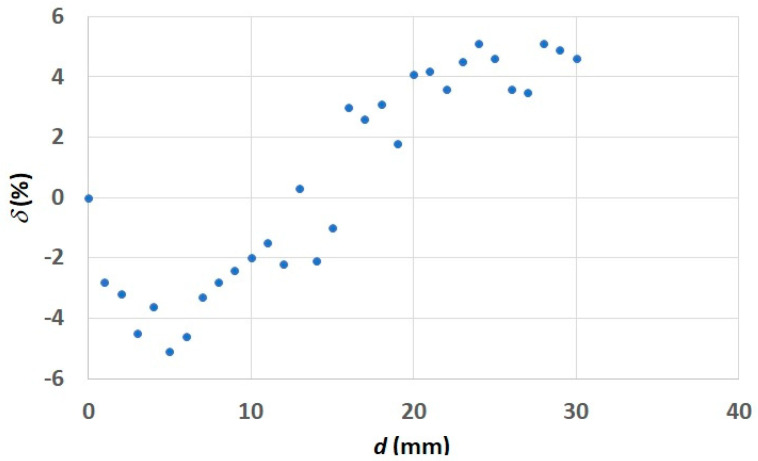
Dependence of the error *δ* as a function of the distance between the lens and the observed object *d*.

In the entire tested range of temperature measurements, the error value did not exceed 1%. It resulted from the fact that the dependence according to which the stand was scaled was also based on the abovementioned function of *ϑ_s_* = f(*R*). As a result of correctness tests run for the reading of distance *d*, the dependence of *δ* = f(*d*) was plotted (Figure 8).

**Figure 9:**
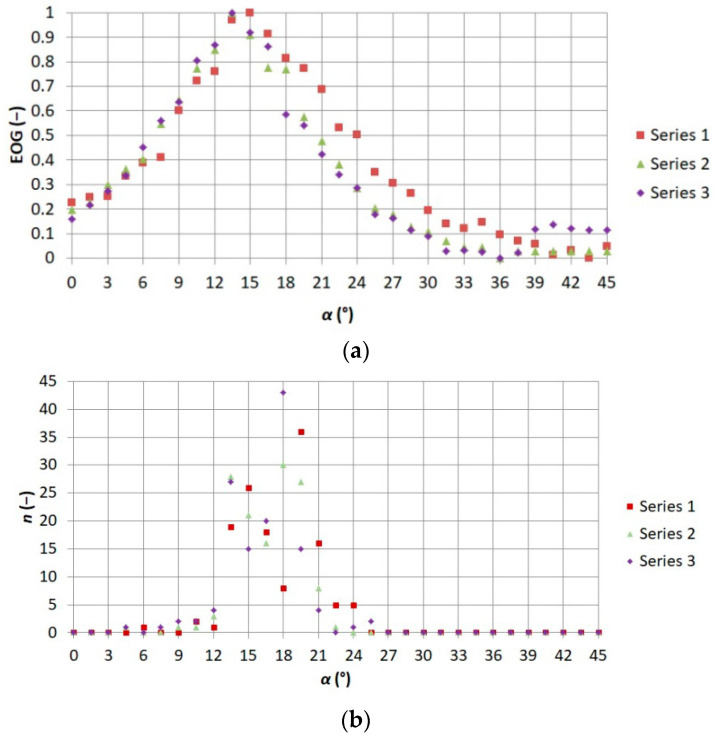
Comparison of relationship (**a**) between EOG (Energy of Gradient) measure of sharpness and the angle of focus adjustment ring placed on the thermographic camera lens *α* 1–3 and (**b**) between numbers of thermograms indicated by observers as sharp n and adjustment ring angle on the thermographic camera lens *α* [19].

In order to determine the influence of the sharpness of the recorded thermogram on the thermographic error, the measure of sharpness based on the change of derivatives in both directions (vertical and horizontal), EOG (Energy of Gradient), was used [17,19]. The results obtained with the mathematical measure of sharpness were normalized. The observers’ responses were compared with the results obtained with the use of the EOG. The obtained results are shown in Figure 9.

**Figure 10:**
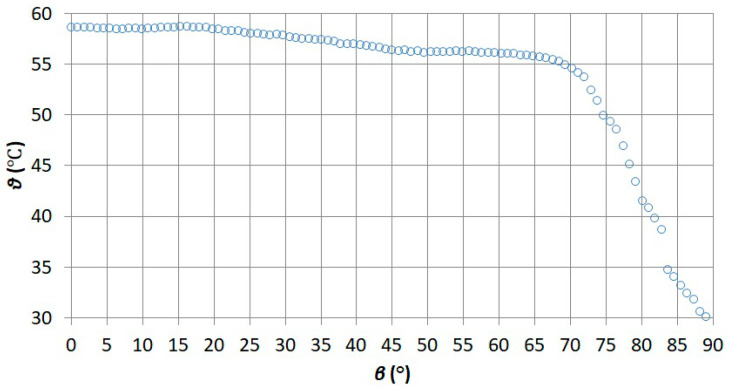
Dependence of the camera indication *ϑ* on the observation angle *β*.

The dependence of the camera indication *ϑ* on the viewing angle *β* obtained as a result of the tests is shown in Figure 10.

Based on the calculations, the measurement uncertainty was determined. The expanded uncertainty of the proposed solution is 1.11 °C for the temperature of the observed object equal to 41.36 °C [47].

## 4. Discussion

The aim of this study was to perform a measuring stand that enables the assessment of the influence of selected factors on the accuracy of the thermographic measurement o thef electronic elements with the use of a macrolens. The results shown in Figure 8 were prepared in order to check the correctness of the setting of *d* on the constructed stand. Analyzing the presented results, it can be concluded that the *d* values set and read by the HMI are correct. This is also confirmed by the results of earlier research [19], during which the same stand was used.

When analyzing the graphs shown in Figure 9, it can be seen that for each of the three series, the maximum sharpness was obtained at a similar value of α. In addition, the values of the maximum sharpness determined by the mathematical measure of sharpness and on the basis of the observers’ indications were observed at a similar value of *α*. This is in line with previous research [17]. This proves that the mathematical measure of sharpness used in this study is correct. Based on the results presented in Figure 9 it can be said that the part of the control system responsible for controlling the stepper motor to adjust the value of *α* works correctly too.

By observing the obtained dependence of the camera indication *ϑ* on the observation angle *β* (Figure 10), it was noticed that the obtained dependence was consistent with the dependence that could be found in the literature [48]. The obtained values are due to the phenomenon of angular emissivity [49]. Based on the comparison of the obtained value with the value from the literature [48], it was found that the value of *β* was correctly indicated on the HMI panel.

The presented stand for short distance thermographic tests was made with many restrictions to ensure that the impact of factors influencing the indication of the IR camera should be minimal. In addition, it is possible to evoke, in a controlled way, only one of the factors in order to verify its influence on the error of temperature measurement with a thermographic camera. Maintaining the stability and high resolution of the settings of individual parameters of the presented stand makes it an innovative solution in the diagnostics of electronic components. Furthermore, due to the use of a PLC controller and HMI panel as management systems, this article becomes more attractive and interdisciplinary. The proposed solution is another example of the unconventional use of a PLC controller commonly associated with industrial automation.

The tests carried out with the use of the presented microscopic thermography stand confirm the correctness of its operation and allow us to create a database on the basis of which it has been possible to assess the impact of the image sharpness and the viewing angle on the measurement error when a thermographic camera is applied. The technique of thermographic measurements classifies them as non-invasive methods, but in the case of diagnosing electronic components, it is necessary to run the measurements from a short distance, taking into account certain parameters. The constructed test stand enables the control of all these factors. The stand described in the article enables the correct and early detection of emerging anomalies in electronic components, which in turn may minimize the time needed to locate an existing fault. This allows us to avoid damage to the entire device and reduce the repair costs.

## 5. Conclusions

An innovative stand for microscopic thermography has been presented in this article. The reason for the creation of such a stand was to verify the impact of the factors influencing the indication of the IR camera on the error of temperature measurement with microscopic thermography. Factors commonly mentioned in the literature as influencing the thermographic measurements are presented, and special attention is paid to those that are important in microscopic thermography. The proposed stand can be used in precise measurements of microscopic thermography. It may be useful in the thermographic measurement of the temperature of electronic component casings, too. However, one limitation is that this particular solution can only be used with other cameras in the Flir E series. In order to use the solution with other commercial cameras, it is necessary to change (or fit) the camera mounting method applied in this solution. This is a task that needs to be solved in further work.

## Data Availability

Not applicable.
